# Limited congruence in phylogeographic patterns observed for riverine predacious beetles sharing distribution along the mountain rivers

**DOI:** 10.1038/s41598-023-44922-w

**Published:** 2023-10-19

**Authors:** Łukasz Kajtoch, Michał Kolasa, Miłosz A. Mazur, Radosław Ścibior, Krzysztof Zając, Daniel Kubisz

**Affiliations:** 1 Institute of Systematics and Evolution of Animals, Polish Academy of Sciences, Sławkowska 17, 31-016 Kraków, Poland; 2 Institute of Environmental Sciences, Faculty of Biology, Jagiellonian University, Gronostajowa 7, 30-387 Kraków, Poland; 3 Institute of Biology, University of Opole, Oleska 22, 45-050 Opole, Poland; 4 Department of Zoology and Animal Ecology, University of Life Sciences in Lublin, Akademicka 13, 20-950 Lublin, Poland; 5 Lądek Zdrój, Poland

**Keywords:** Biogeography, Population dynamics, Population genetics

## Abstract

Riverine predacious beetles (RPB) (Carabidae, Staphylinidae) are highly diverse and numerous elements of riverine ecosystems. Their historical and contemporary distribution and diversity are highly dependent on natural flow regimes and topography of watercourses. Despite broad knowledge of their ecology, data on population genetic diversity and connectivity are lacking. This study aimed to fill this gap in order to solve two principal hypotheses assuming (i) congruence of phylogeographic patterns observed for RPB indicating that they share a common history and the ecological adaptations to the dynamic environment, (ii) genetic structuration of populations according to river basins. The Carpathian populations of four ground beetles and three rove beetles were examined using cytochrome oxidase and arginine kinase sequencing. There are substantial differences in RPB demographic history and current genetic diversity. Star-like phylogeny of *Bembidion* and complex haplotype networks of *Paederus/Paederidus*, with some haplotypes being drainage-specific and others found in distant populations, indicate a general lack of isolation by distance. Signs of recent demographic expansion were detected for most RPB with the latest population collapse for some rove beetles. To some extent, migration of examined species has to be limited by watersheds. Observed phylogeographic patterns are essential for correctly understanding RPB meta-population functioning.

## Introduction

Knowledge on the biogeography of European taxa is the most complete and comprehensive thanks to numerous phylogeographic research on various species^1,2^. It is well known how Mediterranean, temperate and boreal taxa responded to the Pleistocene glacial periods, as well as current constraints for European taxa distribution and diversity^3^. There are several paradigms being well supported by multiple sources of data, which describe refugia identified mostly in the south of the continent, but also local (cryptic) refugia that played a role during various periods for particular taxa being associated with some environments (like forests, steppes, marshlands, etc.)^4,5^. However, some taxa could not be easily assigned to these paradigms, due to their extrazonal distribution. Species inhabiting mountain areas belong to such a non-standard taxa, whose distribution is island-like with more or less isolated populations in mountain ranges. Many mountain species are related to either boreal or arctic populations. Such species were widespread during glacial periods, being currently restricted to warm-stage refugia at high altitudes or latitudes^2^. Consequently, such a species usually consists of many phylogenetic lineages of divergence, justifying their treatment as evolutionary units or independent taxa. Other species of extraordinary phylogeographic patterns are those inhabiting linear habitats, e.g., along watercourses. River networks often cross various climatic zones but are isolated by watersheds, which greatly affect the phylogeography of taxa occupying running waters, river channels or floodplains^6,7^. Moreover, the movement of individuals along watercourses often depends on the water flow, which is unidirectional, and many such species could easily disperse downstream but not hardly upstream^8^. This has consequences for their distribution, diversity and dynamics^9^. Probably among the poorest known phylogeography are organisms inhabiting montane river networks, due to their restricted ranges to rivers crossing mountains at selected altitudes^10^. Therefore, it could be expected that such a species should be restricted to only selected watercourses isolated by mountain ranges and strong unidirectional flow^11^. This would imply the existence of many phylogenetic lineages and a highly limited gene flow. On the other hand, it could be expected that the high dynamics of montane rivers (inundations, floods) had to force some evolutionary adaptations to such an unpredictable environment^12^. Consequently, species living along montane watercourses should exist in meta-populations, which imply some level of migration enabling re-colonization of areas being inundated^13^. It is possible that both patterns are not mutually exclusive and some gene flow exists but could be rather restricted within river basins, while not so between watercourses isolated by geographic barriers. Of course, distribution and diversity is different for freshwater taxa (living only in running waters), riverine species (inhabiting river channels), or riparian organisms (associated with floodplains). Moreover, some taxa are dependent on running water their whole life, whereas others only in immature stages, and still others are distributed in the surroundings of watercourses. That different habitat requirements must have a great impact on biogeography.

Current knowledge on the biogeography of river, riverine or riparian organisms in mountain areas (considering Europe only) is incomplete; it is mostly limited only to selected species and focused on particular areas. The best-known groups are freshwater animals, living their whole lifecycles inside rivers, like fishes^6,14^ and crustaceans^7^. Exemplary studies on insects in the Carpathians are only available for riffle beetles (Elmidae)^15^. Studies on riverine species by means of taxa spending part of their life in water (usually immature stages) and next utilizing surrounding habitats are also infrequent for mountains. Studies on salamanders^16^, Trichoptera^17^, Diptera^18,19^, and the beetle *Carabus variolosus* F.^20^, could be assigned to this type. In particular, riparian taxa meaning those that live along watercourses but are not directly dependent on freshwaters. Research on amphibians^21^ and a study on the beetle *Liparus glabrirostris* could be listed here^22^.

There are some biogeographic patterns that are common for species living along montane watercourses. When restricting this issue to Central European mountain systems, the following regularities arise. Many species living in or along watercourses survived the Pleistocene in local glacial refugia like the southern slopes of the Alps, Dinaric Mts., the Carpathians (e.g.,^14,15,22^). Some taxa have mixed ancestry, originating from Mediterranean mountains and local refugia, and the pathways of their expansion are complex^7,17^. This leads, in some cases, to the formation of hybrid zones across mountain ranges^20,21^. Genetic diversity and spatial structure of populations of riverine or riparian species in mountains reflects their history and contemporary gene flow. For many taxa, distinct evolutionary units were reported from particular mountain ranges, some of which occurred to be of taxonomic values^7,19^. There are also signs of dispersal being restricted by barriers (more pronounced for freshwater than riparian taxa)^6^.

The Carpathians are one of the largest mountain systems on the European mainland^23^, which is known as an important biodiversity hotspot^24^, where numerous evolutionary processes are at play^25,26^. This mountain system is also important due to the naturalness of various habitats^27^. Also, river networks in the Carpathians are of relatively high quality, at least compared to many rivers from the lowlands being heavy transformed. The Carpathians are crossed by numerous rivers belonging to either the Black Sea basin or to the Baltic Sea basin^28^. The connectivity of rivers within the basins is also complex due to numerous watersheds constituting barriers, especially as the Carpathian arch is nearly circular and rivers in inner areas (adjacent to the Pannonian Basin) are isolated by high mountains from outer areas, even if these rivers finally meet the sea. The Carpathians are also an interesting area for studying the biogeography of riverine species, as there is great diversity of rivers flowing at various altitudes and being fed mostly by rainfall, which often cause inundations and occasional floods^29^.

Among the riverine predacious beetles (RPB), the most important ecological groups are ground beetles (Carabidae) and rove beetles (Staphylinidae). Some species belonging to these families are known to be strictly associated with sand, gravel or cobblestone alluvium, however they do not live in the water^30^. These species prefer the initial conditions of river channels, with scattered pioneer vegetation and many potential prey, such as eggs of insects and other invertebrates, fly larvae, mites or numerous Apterygota, as well as small adult insects^31^. Such habitat is described as “alpine rivers and the herbaceous vegetation along their banks,” being protected under the EU habitat directive (Habitat Code: 3220). Because of their narrow environmental requirements, large species diversity, numerous populations and relatively easy methods of collection in the field, these beetles have been designated as indicators for river channel quality^32–34^. Many studies utilize RPB, mostly *Bembidion* carabids^35^, but also other ground and rove beetles^36^ for the assessment of river channel naturalness or the impact of man-made alterations (like damming or channelization). Knowledge on the details of their biology and ecology is available only for some groups and population dynamics in most species are poorly understood, whereas many aspects of population genetics and phylogeography are almost completely unknown^37,38^. Regarding genetic data, only sequences of barcodes are available for some ground and rove beetles (e.g. a barcode library for *Bembidion* species^39^). Mitochondrial markers were also available for some taxa being the subject of phylogenetic studies, however mostly in deep phylogenies of beetles up to the level of families^37^. To our knowledge, there is no study aiming to understand the past and present distribution and diversity of riverine ground or rove beetles using molecular tools.

Filling the gap in the knowledge about the genetic diversity of RPB can be beneficiary for both basic and applied science. Basic because ground and rove beetles are important for understanding the biogeography of riverine species associated with mountain areas as they are top predators in invertebrate communities^38,40^ and applied because genetic data on the populations of these beetles should be implemented in their use as indicators of river channel quality. It is unknown which species have greater or lower genetic diversity and how the connectivity of their population functions. This could have consequences for their use as indicators.

Here we used several species of ground and rove beetles as objects of phylogeographic studies on species living along (sub)montane watercourses in the Carpathians – four species of *Bembidion*: *B. decorum* (Panzer, 1799) (hereafter Bdec); *B. varicolor* (Fabricius, 1803) (hereafter Bvar); *B. modestum* (Fabricius, 1803) (hereafter Bmod); *B. punctulatum* (Drapiez, 1820) (hereafter Bpun), and three species of rove beetles: *Paederus limnophilus* (Erichson, 1840) (hereafter Plim); *Paederidus rubrothoracicus* (Goeze, 1777) (hereafter Prub); *Paederidus ruficollis* (Fabricius, 1781) (hereafter Pruf). Specifically, we aimed to describe the history of RPB, and current diversity of populations (intra- and inter-population genetic variability). This study aimed to verify the following hypotheses:

1. There is substantial congruence of phylogeographic patterns observed for RPB, indicating that they share a common history and that their ecological adaptations to the dynamic environment are similar.
2. Populations of RPB are genetically structured according to basins and being isolated by watersheds.

## Results

### Genetic diversity

After trimming low quality fragments, the lengths of *Cox1* and *ArgK* sequences were 625 bp and 688 bp (Bmod), 637 bp and (Bpun), 636 bp and 674 bp (Bvar), 635 bp and 687 bp (Bdec), 577 bp and 685 bp (Plim), 638 bp and 690 bp (Pruf) and 568 bp and 695 bp (Prub). No indels or stop codons were detected in any sequence.

The overall genetic diversity of all examined beetle species was found to be high (e.g., haplotype diversity calculated for all samples (individuals from all sites) based on both markers is above 0.9 in all taxa) (Table 1). When analyzing the genetic metrics measured for particular regions of the Carpathians and particular river basins, the patterns occurred to be partially concordant among species. The highest values of haplotype and nucleotide diversities were found in the sites located in the Southern Carpathians and high values were also observed in sites from the Eastern Carpathians, as well in some sites from the Western Carpathians, but mostly from the inner (S) site of these mountains. Contrary to that, the lowest diversity metrics were observed in some sites on the outer (N) part of the Western Carpathians, some sites in the outer (E) part of the Eastern Carpathians, and in the Apuseni Mts., but only for some taxa (Table 1).

**Table 1:** Basic statistics based on molecular data (combined cytochrome oxidase subunit I and arginine kinase) calculated for examined predacious riverine beetles in the Carpathians.

Species	*B. modestum*				*B. punctulatum*				*B. varicolor*				*B. decorum*			
Cluster	N	Hnum	Hdiv	Ndiv	N	Hnum	Hdiv	Ndiv	N	Hnum	Hdiv	Ndiv	N	Hnum	Hdiv	Ndiv
Basins																
CarW-O-W	8	1	0	0	13	3	0.410	0.0006	9	5	0.806	0.0010	9	6	0.889	0.0012
CarW-O-C	16	2	0.533	0.0016	19	4	0.380	0.0005	20	5	0.368	0.0012	20	7	0.768	0.0022
CarWE-O-E	6	4	0.867	0.0021	7	4	0.714	0.0023	7	5	0.857	0.0018	7	7	1.000	0.0019
CarW-I-W	–	–	–	–	7	6	0.952	0.0019	13	10	0.923	0.0021	16	10	0.892	0.0025
CarW-I-C	–	–	–	–	4	2	0.667	0.0020	–	–	–	–	–	–	–	–
CarWE-I-W	12	4	0.561	0.0009	16	11	0.908	0.0036	11	7	0.891	0.0022	11	7	0.873	0.0023
CarE-O-N	8	4	0.821	0.0018	8	6	0.929	0.0014	12	10	0.970	0.0021	12	11	0.985	0.0022
CarE-O-E	8	4	0.643	0.0017	4	2	0.500	0.0003	12	9	0.955	0.0021	12	8	0.848	0.0020
CarS-O-S	4	3	0.833	0.0024	8	8	0.0062	0.0043	4	4	1.000	0.0035	8	7	0.954	0.0019
CarS-O-W	–	–	–	–	11	4	0.691	0.0017	8	2	0.536	0.0001	7	2	0.286	0.0004
CarC-I-S	–	–	–	–	7	2	0.476	0.0007	12	4	0.758	0.0017	13	6	0.718	0.0035
CarC-I-A	10	2	0.200	0.0001	13	6	0.872	0.0019	3	1	0	0	11	4	0.691	0.0010
Regions																
W Carpathians	30	9	0.855	0.0027	50	16	0.771	0.0018	49	22	0.855	0.0018	52	27	0.940	0.0029
E Carpathians	28	21	0.971	0.0023	28	17	0.921	0.0027	35	20	0.965	0.0023	35	23	0.948	0.0023
S Carpathians	4	3	0.833	0.0025	19	12	0.901	0.0030	21	7	0.781	0.0019	23	12	0.779	0.0027
Apuseni Mts	10	4	0.733	0.0010	20	8	0.889	0.0019	6	2	0.600	0.0014	16	6	0.742	0.0014
All	72	37	0.966	0.0024	117	47	0.910	0.0034	111	45	0.945	0.0024	126	62	0.957	0.0028

Species	*P. limnophilus*				*P. ruficollis*				*P. rubrothoracicus*							
Cluster	N	Hnum	Hdiv	Ndiv	N	Hnum	Hdiv	Ndiv	N	Hnum	Hdiv	Ndiv				
Basins																
CarW-O-W	9	7	0.917	0.0034	–	–	–	–	4	4	1.000	0.0028				
CarW-O-C	20	6	0.705	0.0029	20	6	0.621	0.0015	19	4	0.298	0.0008				
CarWE-O-E	8	4	0.750	0.0027	8	6	0.929	0.0034	8	4	0.750	0.0028				
CarW-I-W	15	11	0.943	0.0034	4	3	0.833	0.0015	16	12	0.917	0.0055				
CarW-I-C	6	6	1.000	0.0039	5	3	0.700	0.0020	–	–	–	–				
CarWE-I-W	18	13	0.954	0.0059	12	10	0.955	0.0030	16	15	0.992	0.0046				
CarE-O-N	6	2	0.600	0.0033	9	8	0.972	0.0026	3	3	1.000	0.0042				
CarE-O-E	4	1	0.000	0.0000	12	9	0.955	0.0029	12	10	0.970	0.0091				
CarS-O-S		–	–	–	–	7	1.000	0.0037	8	7	0.964	0.0078				
CarS-O-W	3	3	1.000	0.0085	7	5	0.905	0.0017	10	8	0.956	0.0094				
CarC-I-S	7	7	1.000	0.0129	10	2	0.467	0.0004	9	7	0.917	0.0060				
CarC-I-A	12	10	0.955	0.0098	11	3	0.564	0.0004	13	11	0.974	0.0075				
Regions																
W Carpathians	54	29	0.946	0.0036	33	16	0.856	0.0029	39	20	0.825	0.0032				
E Carpathians	32	16	0.938	0.0061	37	27	0.976	0.0032	39	32	0.987	0.0062				
S Carpathians	10	10	1.000	0.0122	21	11	0.819	0.0021	27	21	0.980	0.0081				
Apuseni Mts	12	10	0.955	0.0098	14	4	0.703	0.0006	13	11	0.974	0.0075				
All	108	64	0.976	0.0069	105	53	0.956	0.0032	118	79	0.973	0.0062				

N – sample size, Hnum – haplotype number, Hdiv – haplotype diversity, Ndiv – nucleotide diversity. Names of clusters according to Table 1.

### Spatial genetics

**Figure 1:**
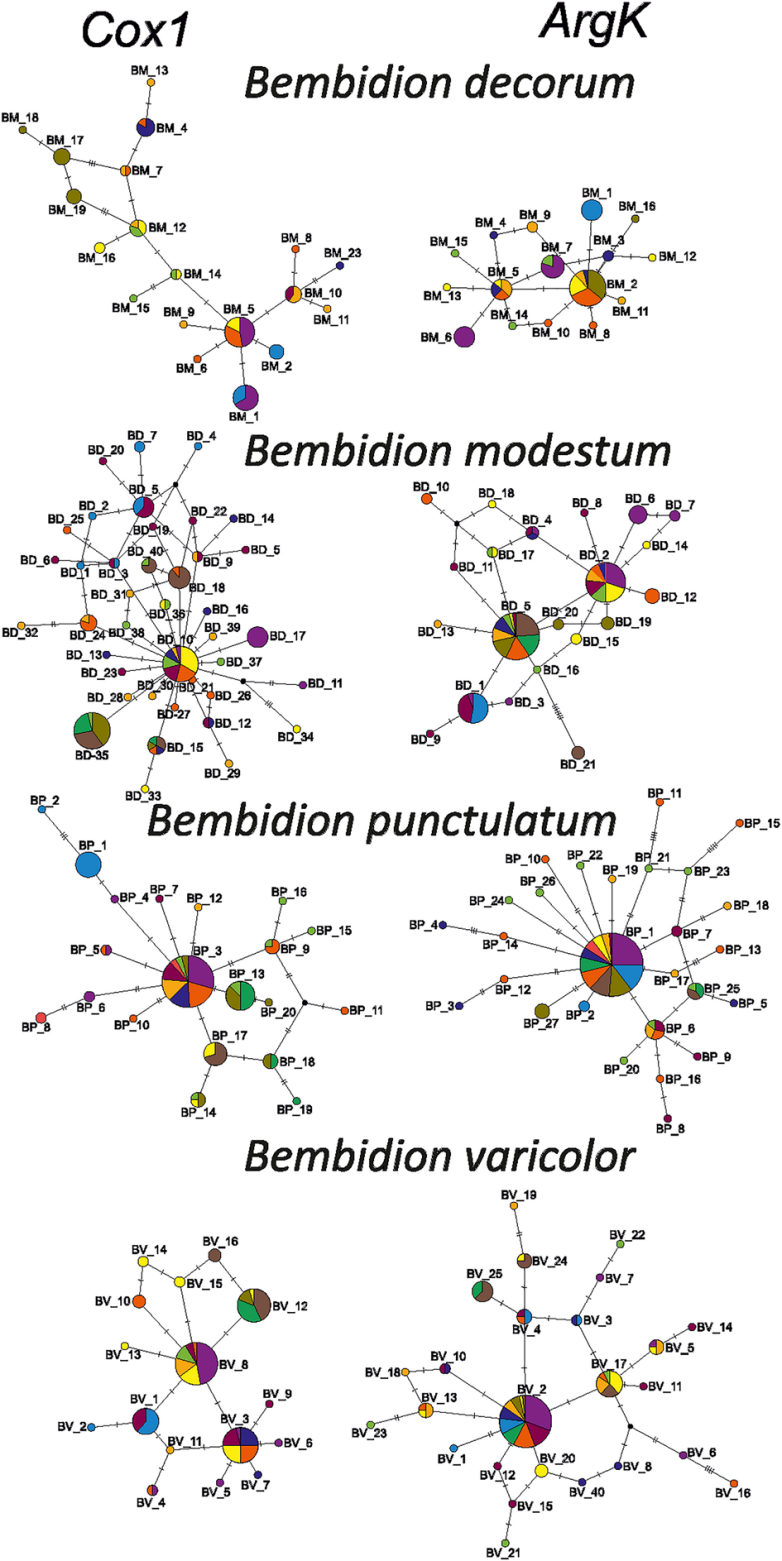
Haplotype networks constructed for cytochrome oxidase I gene (*Cox1*) and arginine kinase gene (*ArgK*) for populations of examined *Bembidion* beetles in the Carpathians. Colors correspond to defined river basins (for details see Table 1 and Figure 6).

**Figure 2:**
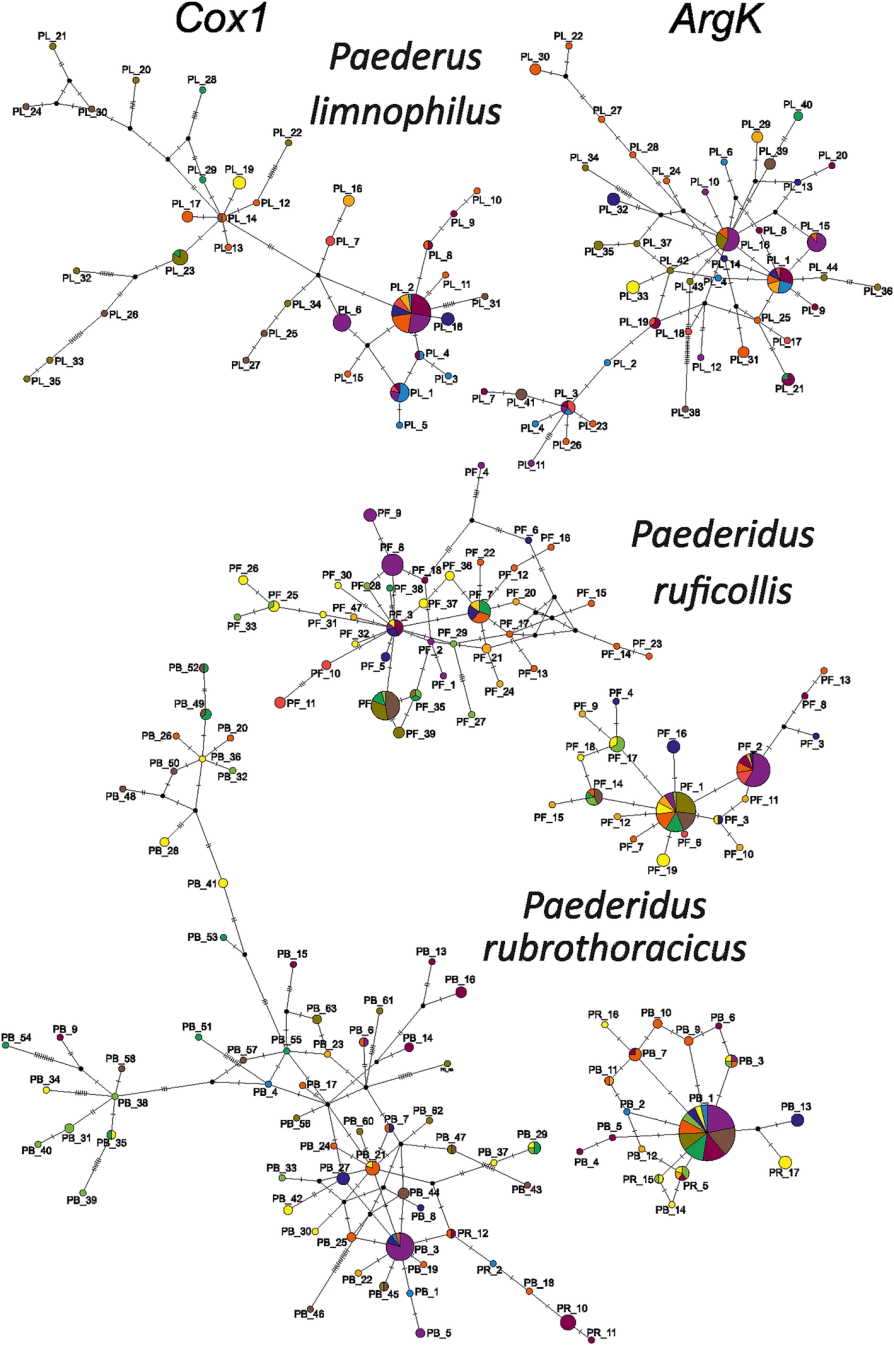
Haplotype networks constructed for cytochrome oxidase I gene (*Cox1*) and arginine kinase gene (*ArgK*) for populations of examined *Paederus* and *Paederidus* beetles in the Carpathians. Colors correspond to defined river basins (for details see Table 1 and Figure 6).

**Figure 3:**
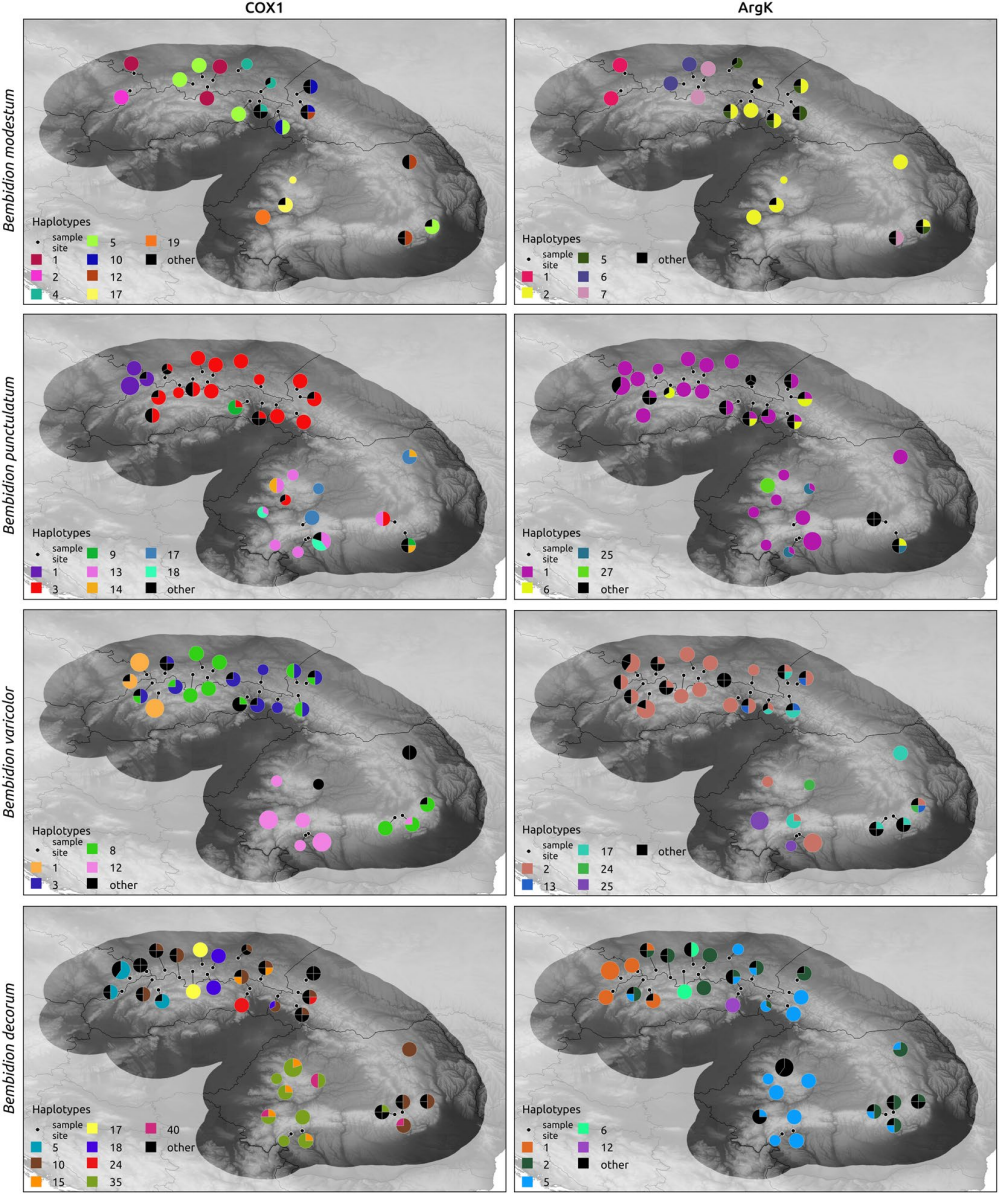
Maps presenting the distribution of the most common haplotypes of two markers (*Cox1* – cytochrome oxidase subunit I and *ArgK* – arginine kinase) for populations of examined *Bembidion* beetles in the Carpathians. Black represents all other remaining (less frequent) haplotypes. Maps created with use of QGIS ver. 3.28.9 (http://qgis.org) and CorelDraw ver. 18 (http://coreldraw.com).

**Figure 4:**
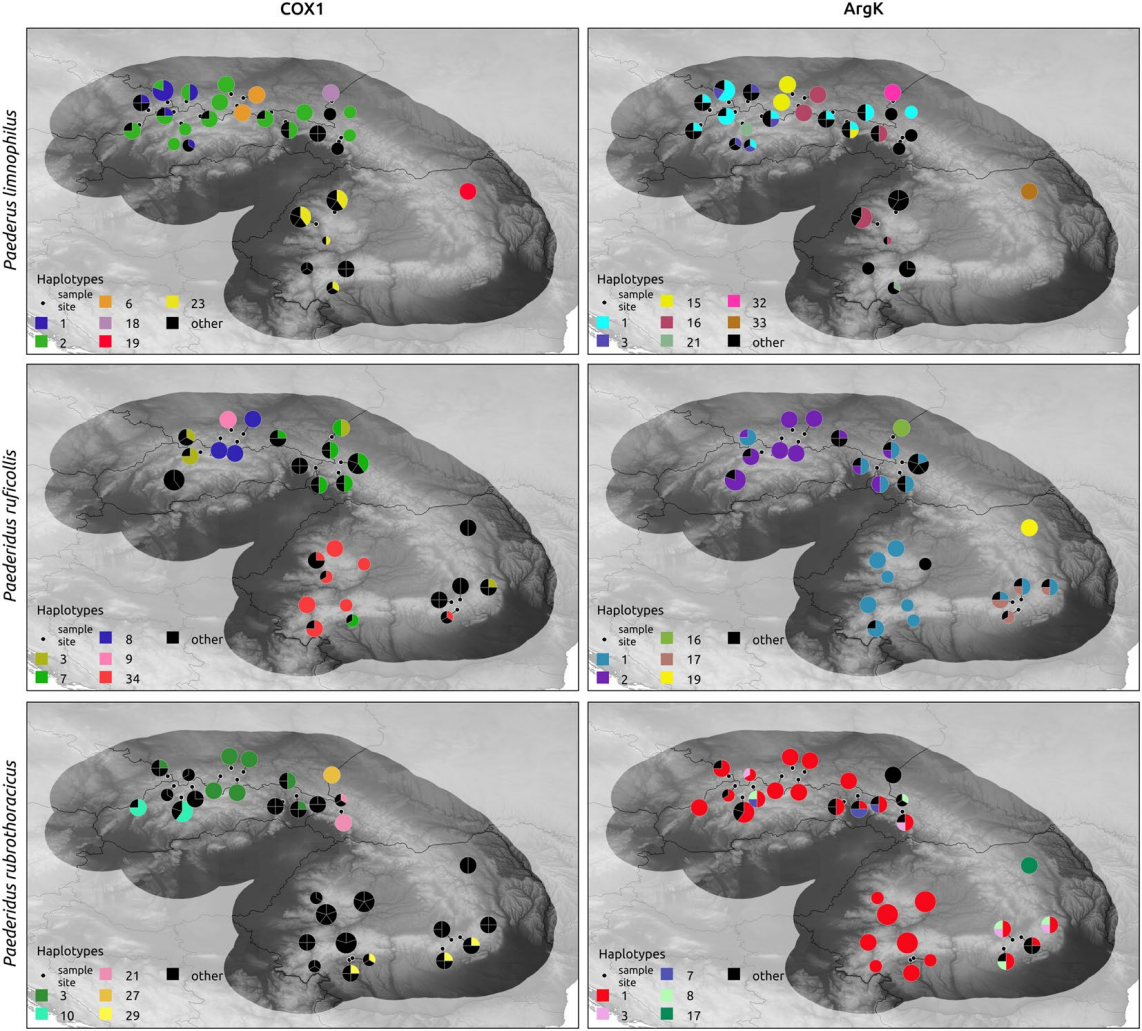
Maps presenting the distribution of the most common haplotypes of two markers (*Cox1* – cytochrome oxidase subunit I and *ArgK* – arginine kinase) for populations of examined *Paederus* and *Paederidus* beetles in the Carpathians. Black represents all other remaining (less frequent) haplotypes. Maps created with use of QGIS ver. 3.28.9 (http://qgis.org)  and CorelDraw ver. 18 (http://coreldraw.com).

Haplotype networks generated on two markers (*Cox1* and *ArgK)* resulted in very complex patterns, with some haplotypes being common for more beetles, and this was more pronounced in the case of the *Bembidion* than the *Paederus/Paederidus* taxa (Figs. 1 and 2). Star-like phylogenies were observed mostly for ground beetles in single-marker networks, whereas spatial-genetic patterns for rove beetles were more complex, with many haplotypes found in a single or a few individuals. Despite the complexity of haplotype networks, some haplotypes were found to be specific for particular river basins or regions of the Carpathians, indicating some degree of spatial structuring (Figs. 3 and 4). Consistency between phylogeographic patterns observed for *Cox1* and *ArgK* was limited, as only some populations occurred to be distinct with respect to both markers (Figs. 3 and 4). This was additionally confirmed by patterns of *G*_*ST*_ and *F*_*ST*_ indices among populations calculated for both markers separately.

The heatmaps of *F*_*ST*_ and *G*_*ST*_ indices showed complex patterns of population differentiation and gene flow level (Fig. S1). Generally differentiation of populations of row beetles occurred to be lower than ground beetles. Moreover, values of *F*_*ST*_ and *G*_*ST*_ indices were larger for *Cox1* than for *ArgK* markers. The highest genetic differences occurred between geographically most distant populations.

That limited spatial structuring was further confirmed by AMOVA and the Mantel test. Molecular variance measured between four major regions of the Carpathians (Western vs. Eastern vs. Southern vs. Apuseni Mts.) revealed a relatively low proportion of variance, which could be attributed among these regions (from only 6% in Bpun to 34% in Bmod; Table S1). These values were only slightly higher when dividing the population according to defined river basins (from 21% in Bvar to 50% in Plim; Table S1). The majority of molecular variance, in both ways of grouping, occurred to be within populations (from 27% in Bmod to 68% in Prub for regions and from 20% in Plim to 69% in Prub for basins; Table S1).

The Mantel test showed that IBD is significant but weak only for Bpun (R = 0.08; *p* = 0.046). For Bvar, IBD was also significant but negative indicating that distant populations are even slightly less distinct than adjacent populations (R = − 0.13; *p* = 0.006). For other species, IBD was insignificant: Bmod (R = − 0.11; *p* = 0.833), Bdec (R = 0.03; *p* = 0.335), Plim (R = − 0.03; *p* = 0.708), Pruf (R = − 0.00; *p* = 0.567), and Prub (R = − 0.14; *p* = 0.996) (Fig. S2).

### Demography

Tajima’s D test was negative for all species but significant only in the case of Bpun and Plim (Table S2). On the other hand, Fu’s Fs test was also negative and significant for all species (Table S2). Consequently, both tests indicated population size expansion (e.g., after a bottleneck or a selective sweep), at least for some of the examined species.

**Figure 5:**
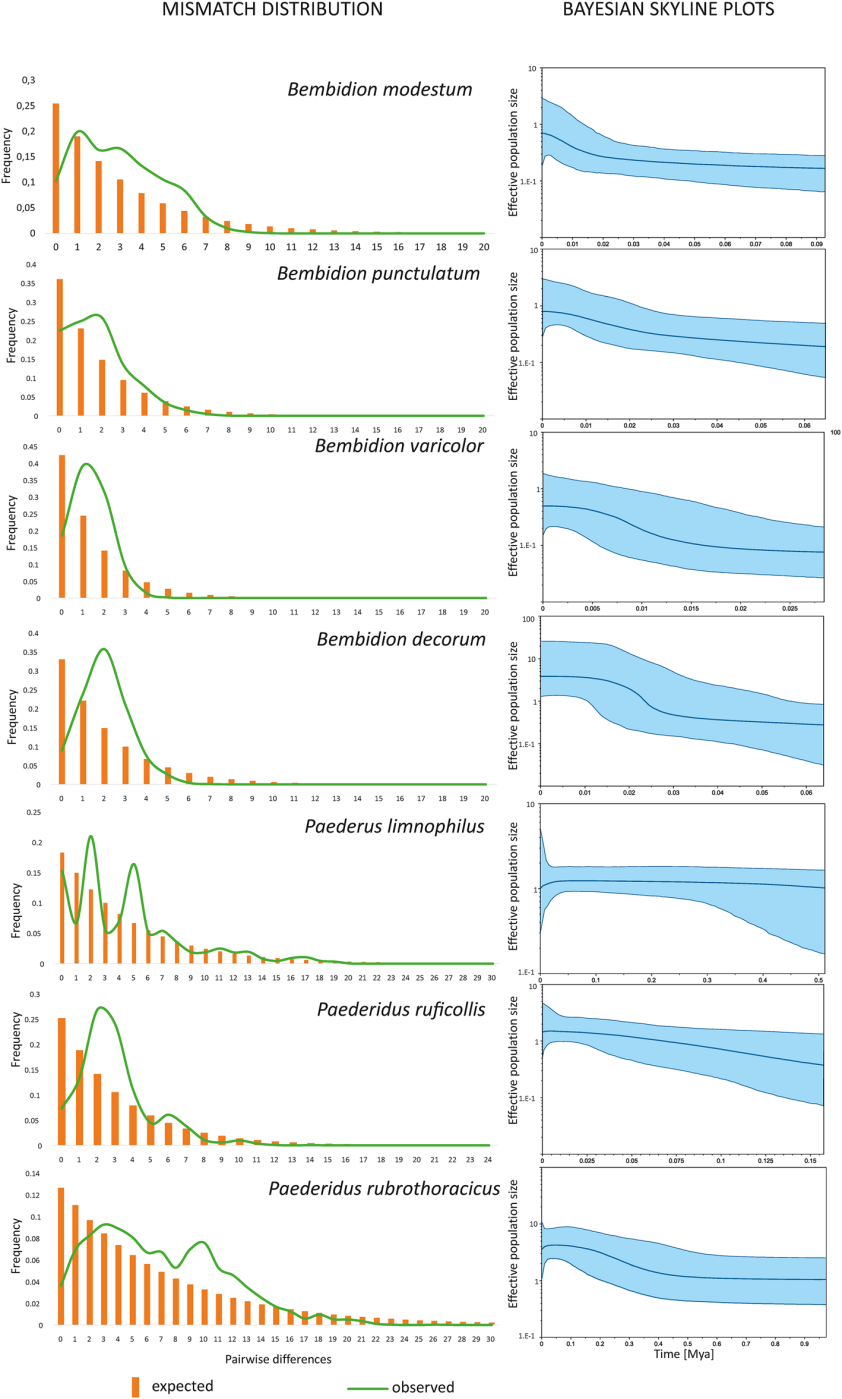
Visualization of demographic statistics measured for populations of examined predacious riverine beetles in the Carpathians. Left panel—histograms of mismatch distribution. Right panel—results of Bayesian skyline plot analyses.

MD occurred to be unimodal and left-skewed for Bpun, Bvar, Bdec and Pruf, whereas it was multimodal and more right-skewed for Bmod, particularly for Plim and Prub (Fig. 5). Estimates of demographic events suggested a consistent period of expansion times for four out of seven species (Bmod, Bvar, Bdec, Pruf) in a range between 12 and 9 Kya. For Bpun, these estimates were a little older (16–13 Kya), whereas for two species they were much older: Plim (27–22 Kya) and Prub (45–36 Kya).

BSP indicated that all examined species underwent changes in their effective population sizes (Fig. 5). Three species (Bmod, Bpun, Pruf) showed similar patterns of constant but slow growth in population size; two species (Bvar and Bdec) a more stepped growth of population sizes around 20 Kya (Bdec) or 10 Kya (Bvar). Prub also grew in population size but recently (0.3–0.2 Kya) its population collapsed in size. Finally, Plim showed a relatively stable trend with a very recent reduction in population size (Fig. 5).

## Discussion

### History

Demographic analyses on genetic data lead to a concordant picture of the past distribution of RPB in the Carpathians. Genetic data showed that expansion of the examined beetles is rather a case of recent times, with some exceptions. Demographic estimates pointed to some different recent histories of particular taxa. Most RPB appeared to increase in population size since end of the last glaciation, however some taxa (Bpun, Prub) probably started expansion earlier—during LGM. Results for Plim are inconsistent, as mismatch distribution suggest its earlier expansion, whereas the Bayesian skyline plot shows a relatively stable population size. Only for two taxa (Plim and Prub) are there signs of a very recent decline of population sizes, however, confidence intervals are rather wide; therefore, this finding needs to be taken with caution. It seems that the Carpathians were not an important refugium for RPB, contrary to some other species living in watercourses^6,7,14,15^. Cold-adapted freshwater taxa living in mountains possibly had more stable environment even during Pleistocene in rivers in the Carpathians, whereas species living on alluvium could be more susceptible to unfavorable climatic conditions in glacial periods.

### Current diversity

The southern mountain ranges of the Carpathians harbors, on average, populations having highest values of diversity indices. This is probably an effect of their origin or rather the direction of expansion in the Carpathians from the south to the north during the Holocene period. This is confirmed by the lowest genetic diversity from the outer part of the Western Carpathians, settled being probably at the end of beetle spread in the Holocene period. The Southern Carpathians are known as an important local glacial refugium for temperate taxa in Europe^1–5^. The existence of most (genetically) diverse populations of RPB in this part of the Carpathian range reflects that common phylogeographic pattern, although rather recent estimates of expansion of these beetles in the Carpathians prevent from drawing conclusions about the role of the southern ranges as their glacial refugia. To solve this issue, further studies including populations from the Balkans, the Dinaric Mts., and the Alps are needed, as it is likely that RPB settled the Carpathians from external refugia. Interesting is that some populations in the Eastern and southern slopes of the Western Carpathians possess also high genetic diversity. This could be explained by the settlement of these areas by diverse populations or by beetles from different sources (various migration routes and/or waves). This issue could not be investigated based on selected markers, and genomic information should be implemented.

Highly complex patterns of haplotype networks, both generated from both mtDNA and nuclear genes, are interesting. This is contrary to freshwater invertebrates with known phylogeography in the Carpathians (e.g., gammarids)^7,15^ or riparian insects (e.g., *Liparus glabrirostris*)^22^. For these other animals associated with mountain watercourses, many phylogenetic lineages were determined across the Carpathians, being mostly geographic-specific, which indicate spatial structuring. Mitochondrial haplotype networks obtained for *Bembidion* beetles, but not for rove beetles, are quite similar to those observed in Elmidae^15^, for which common haplotypes were found in many sites, indicating limited spatial structuring. RPB seemed to not be clearly structured genetically over the Carpathians. Only a portion of molecular variance could be attributed between regions or basins. Only for two out of the seven taxa, a significant correlation between geographic and genetic distances was detected, however always being very weak. This is caused by many haplotypes found in single localities and some haplotypes being found in several individuals from distantly localized populations (possible signs of long distance dispersal). On the other hand, a mapping of more common haplotypes showed that some of them are specific to particular areas of the Carpathians, but they are rarely associated with river basins. In summary, these patterns generally reject the assumption that populations of RPB are genetically structured remaining isolated by barriers (watersheds on mountain ranges) and that beetles found in rivers belonging to the same basin are not (or are less) genetically isolated than beetles captured in different basins. It was suspected that RPB are poor dispersers due to their behavior during inundations—rove beetles escape on the ground (although are capable of flight), whereas ground beetles fly very short distances (authors’ observations). This assumption is likely wrong and these species are capable of long distance migration, possibly only during some phases of their lifecycle, e.g., emergence after wintering or during mating^41^. Unfortunately, knowledge on the biology of these species is very limited, with many details on the ecology and biology (e.g. movement, reproduction) remaining unknown^42^. High genetic diversity and complex phylogeographic patterns suggest that predacious riverine beetles exist in a system of meta-populations, with individuals moving between the occupied sites. It is most probable that some populations are vanishing during floods (individuals are either killed by the water or forced to escape)^41^. This could be compared to the genetic diversity of Elmidae^15^. Subsequently, empty sites are colonized by ground and rove beetle individuals from other areas (rivers). This would explain the missing clear spatial structure of their populations, as gene flow had to be substantial, albeit partially limited by distance. These are just possible explanations that need to be verified. Most likely there are some differences in responses of ground and rove beetles to the changeable environment, which resulted in more complex haplotype networks observed for *Paederus/Paederidus* than for *Bembidion*. Likely different mechanisms of isolation are responsible for that, especially as simple isolation by distance is generally absent in RPB, and other isolation models (by dispersal limitations, by adaptation) need to be verified^43^.To solve this issue examination of loci responsible for environmental adaptations is required^44^.

### Implications

Ground and rove beetles, particularly taxa belonging to the examined genera, are known as excellent indicators of river channel quality^32–34,36^. However, their utility as indicators was based solely on their narrow habitat requirements^33,34^. Moreover, these species are used as indicators at the level of community. Such a basic or applied use of these beetles is done without knowledge on the details of the history and current diversity of particular species in the sampled area, which could have important consequences for their distribution, density or dynamics. This study brings some novel findings, which could be valuable for the use of predacious riverine beetles as indicators. First of all, it seems that RPB have many commonalities in their recent (Holocene) spread over the Carpathians, which proves that the history of particular taxa should not be issue in their use in community studies for monitoring or conservation purposes, as apparently, all these beetles experienced similar changes in distribution and abundance. Consequently, their present occurrence has to be mostly shaped by contemporary environmental constraints, rather than past history. Moreover, the majority of their populations across the Carpathians possessed high genetic variability and only some areas seemed to be settled by less genetically diverse populations. This information could also be important for comparing biodiversity data from various areas, as it is possible that populations having lower genetic diversity are more prone to being affected by inundations or man-made alterations of rivers. The open question is whether lower genetic diversity is an effect of origin of populations (historic expansion?) or it is a result of some external factors acting recently like river regulation and damming. This problem could be also solved with use of genomic data in conjunction with landscape characteristics (landscape genetics)^45,46^.

## Conclusions

For the first time, a comprehensive elaboration of the phylogeography of RPB from a mountain range on the example of the Carpathians is presented. Molecular analyses for several species of RPB substantially broaden the knowledge on the biogeography of the Carpathians^25,26^ as far as information about species inhabiting river channels but not living in running waters like crustaceans^7,15^ or fishes^6,14^. Phylogeographic and demographic data revealed many common features of examined beetles being members of two unrelated groups, but sharing environment and belonging to the same trophic guild. Therefore, Hypothesis 1 was confirmed as there is substantial congruence of phylogeographic patterns observed for RPB, indicating that they share a common history and that their ecological adaptations to the dynamic environment are similar.

Higher genetic diversity was reported mostly from the Southern Carpathians, however many sites in the Eastern and inner Western Carpathians also harbor populations of high variability. Populations of these beetles are not clearly geographically structured. However, some common haplotypes occurred to be widespread in particular regions or basins of the Carpathians, indicating some degree of isolation. This is confirmed by weak isolation by distance reported only for some taxa. It seems that the examined beetles are more capable of greater migration than expected based on their limited flight activity. Therefore, Hypothesis 2 was rejected as populations of RPB are genetically structured according to basins and being isolated by watersheds.

The crucial discovery of this study is that species of RPB, regardless of their taxonomic affinity and phylogenetic relations, have many common features describing their phylogeography and demography.

## Material and methods

### Species characteristics and selection

*Bembidion* Latreille, 1802 is the genus which the highest species richness within whole Carabidae family. To date, more than 1200 species have been described^47^. In Palaearctic region occurring more than 900 species and subspecies, while from Europe so far discovered approximately 400 taxa^48^. Most species live along banks of running or standing waters, but also on open areas^37^.

*Paederus* Fabricius 1775 and *Paederidus* Mulsant & Rey, 1877 are members of the same tribe Paederini Fleming, 1821 (Blackwelder, 1939). Approximately 650 species of these genera occur in the world, mostly in subtropical and tropical areas^49^*.* Only some genera are well represented in Europe, very few in northern parts of the continent and/or preferring mountainous habitats. About five species of the genus *Paederidus* are currently known from Europe, while *Paederus* in this area has about 13 representatives. *Paederus* species preferring wet and humid habitats. They live on the edge of reservoirs and rivers, on mudflats, among sparse vegetation, only a few species reside in dry and warm environments. *Paederidus* is also most commonly found on the banks of rivers and streams, with a distinct preference for mountain and foothill sites.

Four species of *Bembidion* were selected for this study: *B. decorum* (Panzer, 1799) (hereafter Bdec); *B. varicolor* (Fabricius, 1803) (hereafter Bvar); *B. modestum* (Fabricius, 1803) (hereafter Bmod); *B. punctulatum* (Drapiez, 1820) (hereafter Bpun).

Three species of rove beetles (Staphylinidae) were selected for this study: *Paederus limnophilus* (Erichson, 1840) (hereafter Plim); *Paederidus rubrothoracicus* (Goeze, 1777) (hereafter Prub); *Paederidus ruficollis* (Fabricius, 1781) (hereafter Pruf).

All mentioned species from both families share the same ecological features. Adult and larval forms of all species live in the same environment and are predatory and simultaneously are not dependent on vegetation, which is highly variable and unstable in the environment of dynamic mountain rivers^38,40^). During the selection of species for the study, we were also guided by the following criteria: (i) all species with mountain-type distribution, known to occur throughout the Carpathian mountain system; (ii) habitat requirements of the species limited to gravel or cobblestone alluvium in (sub)montane river valleys; (iii) numerous populations over the Carpathians; (iv) numerous and rich populations, allowing to catch at least 5–10 specimens from as many species as possible per site. During field sampling (see below), dozens of species of ground and rove beetles were caught and preserved for molecular use. However, most of these taxa were found in only single or several localities from particular parts of the Carpathians, usually as a single individual per site. These species were discarded due to insufficient sampling. Others of a relatively large number of individuals collected occurred to be lowland species with ranges spanning into the Carpathians, and were also discarded.

### Sampling

Beetles were sought and collected across the Carpathians between 2017 and 2021. Unfortunately, due to the Russian invasion of Ukraine, we had to abandon the plan for sampling beetles in the southern part of the Ukrainian Carpathians in 2022 year. That resulted in approx. 200 km gap in sampling design. Although samples already collected in Eastern Carpathians in Poland, Slovakia, Ukraine and Romania represent well this part of beetle ranges. Individuals were sampled from river alluvia using of exhauster. These beetles usually hide under cobblestones or inside gravel. To facilitate finding of individuals the water was flushed on the ground, what forced beetles to move – individuals run from the area being wet as the result of natural escape behavior^42^. Sampling was done during the highest abundance of predacious beetles since end of May to the beginning of July.

**Figure 6:**
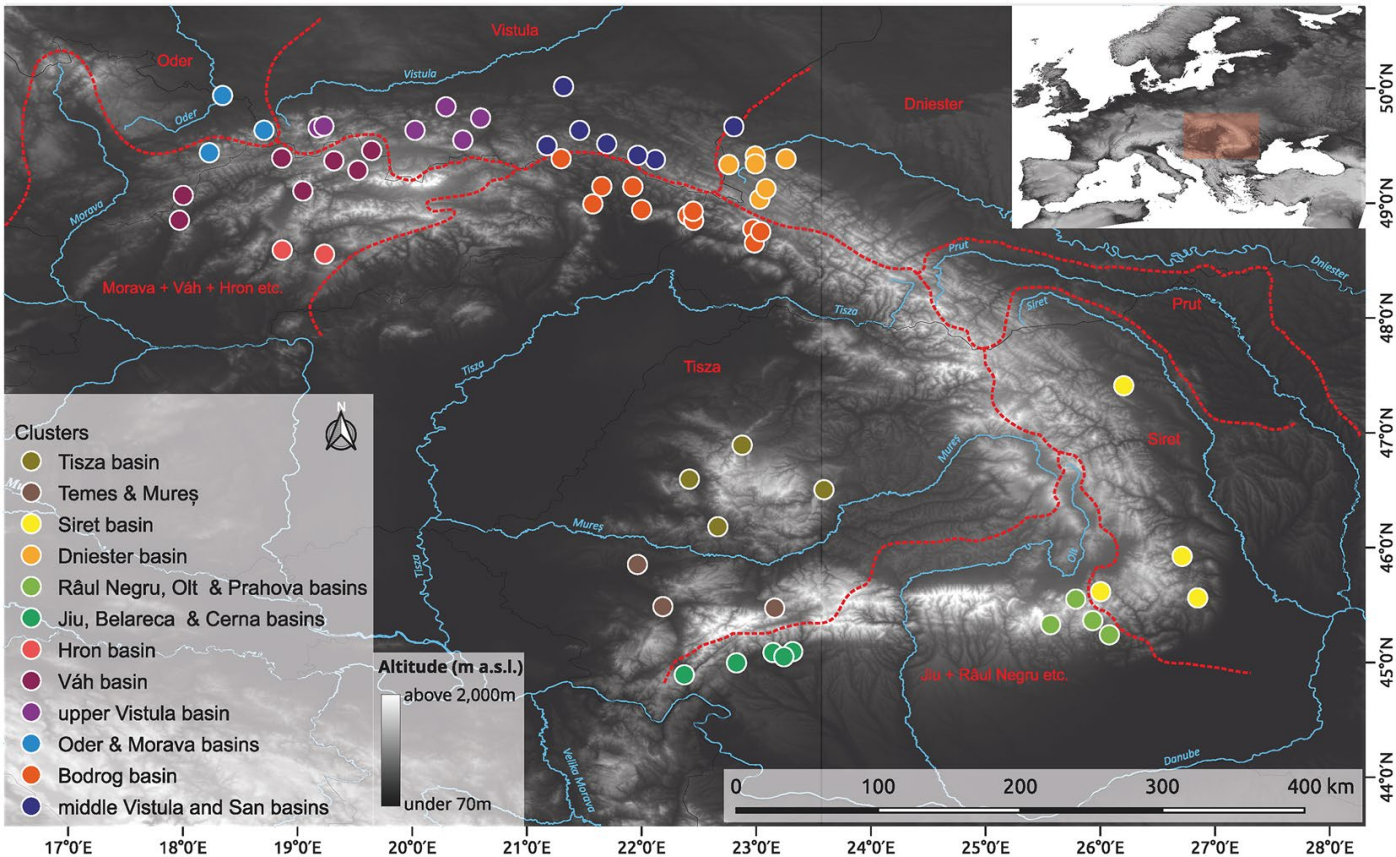
Localization of sampling sites of riverine predacious beetles over the Carpathian range. Each major tributary used in the assignment of sites for analyses is presented in a different colour. Map created with use of QGIS ver. 3.28.9 (http://qgis.org)  and CorelDraw ver. 18 (http://coreldraw.com).

In total we sampled beetles for molecular use on 159 sites (CZ – 9, PL – 50, RO – 42, SK – 36, UA – 22) from 111 (CZ – 7, PL – 37, RO – 31, SK – 22, UA – 15) main rivers and their smaller but always named tributaries (Fig. 6). Particular species were selected from 89 sites (Bvar), 129 (Bdec), 109 (Bpun), 35 (Bmod), 109 (Prub), 82 (Pruf) and 88 (Plim) (Table 2).

**Table 2:** Sampling desing of predacious riverine beetles in the Carpathians for molecular analyses.

Country	Region	River	Tributary	N	E	Altitude	Cluster—symbol	Cluster—description (river basins)	*B. modestum*	*B. punctulatum*	*B. varicolor*	*B. decorum*	*P. limnophilus*	*P. ruficollis*	*P. rubrothoracicus*
									number of examined individuals						
Czechia	W Carpathians	Oder	Oder	49.937924	18.346484	196	CarW-O-W	Oder & Moravia	4	4					
Czechia	W Carpathians	Olše	Oder	49.634444	18.707778	328	CarW-O-W	Oder & Moravia		4	5	4	5		4
Czechia	W Carpathians	Bečva	Morava	49.438889	18.230833	446	CarW-O-W	Oder & Moravia	4	5	4	5	4		
Poland	W Carpathians	Soła	Vistula	49.6569889	19.1793191	361	CarW-O-C	upper Vistula					4	4	
Poland	W Carpathians	Koszarawa	Vistula	49.670416	19.229339	363	CarW-O-C	upper Vistula		3	4	4			3
Poland	W Carpathians	Raba	Vistula	49.634036	20.024089	424	CarW-O-C	upper Vistula	4	4	4	4	4	4	4
Poland	W Carpathians	Stradomka	Vistula	49.8397274	20.2942635	242	CarW-O-C	upper Vistula	4	4	4	4	4	4	4
Poland	W Carpathians	Dunajec	Vistula	49.548886	20.441319	354	CarW-O-C	upper Vistula	4	4	4	4	4	4	4
Poland	W Carpathians	Łososina	Vistula	49.7413596	20.5962235	265	CarW-O-C	upper Vistula	4	4	4	4	4	4	4
Poland	W Carpathians	Kłopotnica	Vistula	49.6337356	21.4592744	272	CarWE-O-E	middle Vistula and San						4	
Poland	W Carpathians	Zdynia	Vistula	49.4982635	21.1750748	421	CarWE-O-E	middle Vistula and San			4				
Poland	W Carpathians	Jasiołka	Vistula	49.516111	21.696389	343	CarWE-O-E	middle Vistula and San							4
Poland	W Carpathians	Wisłoka	Vistula	50.016102	21.318711	186	CarWE-O-E	middle Vistula and San	3	4		3			
Poland	E Carpathians	Osława	San	49.378889	22.119444	414	CarWE-O-E	middle Vistula and San					4		
Poland	E Carpathians	Wisłok	San	49.416389	21.965556	226	CarWE-O-E	middle Vistula and San	3	3	3	4			
Ukraine	E Carpathians	Vihor	San	49.665648	22.804710	270	CarWE-O-E	middle Vistula and San					4	4	4
Czechia	W Carpathians	Vlára	Váh	49.067500	18.003333	250	CarW-I-W	Váh				4	4		
Slovakia	W Carpathians	Lipnica	Váh	49.458522	19.646589	600	CarW-I-W	Váh		3		4			
Slovakia	W Carpathians	Drietomica	Váh	48.852222	17.969722	195	CarW-I-W	Váh							4
Slovakia	W Carpathians	Bystrica	Váh	49.3927749	18.8623673	402	CarW-I-W	Váh			4	4	4		3
Slovakia	W Carpathians	Váh	Váh	49.1064831	19.0461648	398	CarW-I-W	Váh		4	5	4	3		5
Slovakia	W Carpathians	Studeny	Váh	49.286150	19.526089	616	CarW-I-W	Váh			4		4	4	
Slovakia	W Carpathians	Bila Orava	Váh	49.368839	19.316555	670	CarW-I-W	Váh							4
Slovakia	W Carpathians	Handlovka	Hron	48.591389	18.866667	254	CarW-I-C	Hron		4			3	5	
Slovakia	W Carpathians	Slatina	Hron	48.555278	19.235278	331	CarW-I-C	Hron					3		
Slovakia	W Carpathians	Kamenec	Bodrog	49.38741	21.2976864	351	CarWE-I-W	Bodrog					4		
Slovakia	W Carpathians	Topľa	Bodrog	48.9937821	21.5751221	145	CarWE-I-W	Bodrog		4	4	4			4
Slovakia	E Carpathians	Laborec	Bodrog	49.143333	21.923889	231	CarWE-I-W	Bodrog	4		4				4
Slovakia	E Carpathians	Ondava	Bodrog	49.145278	21.653333	278	CarWE-I-W	Bodrog	4			4	4		
Slovakia	E Carpathians	Cirocha	Bodrog	48.943611	22.000556	165	CarWE-I-W	Bodrog		4				4	
Slovakia	E Carpathians	Ublya	Bodrog	48.894722	22.402222	204	CarWE-I-W	Bodrog		4				4	
Ukraine	E Carpathians	Uz	Bodrog	48.850278	22.453611	183	CarWE-I-W	Bodrog	4				4		
Ukraine	E Carpathians	Ulichka	Bodrog	48.929722	22.449444	221	CarWE-I-W	Bodrog			3	3			4
Ukraine	E Carpathians	Zhdenivka	Bodrog	48.775278	22.969722	412	CarWE-I-W	Bodrog						4	
Ukraine	E Carpathians	Mala Pynya	Bodrog	48.653333	22.983056	305	CarWE-I-W	Bodrog		4		4	3		
Ukraine	E Carpathians	Latorica	Bodrog	48.751389	23.038333	400	CarWE-I-W	Bodrog					3		4
Ukraine	E Carpathians	Linynka	Dniester	49.410289	22.991194	352	CarE-O-N	Dniester			4				
Ukraine	E Carpathians	Mszanka	Dniester	49.335713	22.760733	554	CarE-O-N	Dniester					3	4	
Ukraine	E Carpathians	Gnila	Dniester	49.035833	23.030278	599	CarE-O-N	Dniester			4	4		5	3
Ukraine	E Carpathians	Dniester	Dniester	49.3419164	22.9914808	397	CarE-O-N	Dniester	4	4					
Ukraine	E Carpathians	Stryj	Dniester	49.125556	23.084722	558	CarE-O-N	Dniester	4	4					
Ukraine	E Carpathians	Bystrytsia	Dniester	49.385833	23.258611	341	CarE-O-N	Dniester			4	4	3		
Romania	E Carpathians	Râmnic/Siret	Siret	45.562985	26.848629	318	CarE-O-E	Siret	4						
Romania	E Carpathians	Moldova	Siret	47.409722	26.203333	363	CarE-O-E	Siret	4	4	4	4	4	4	4
Romania	E Carpathians	Buzău	Siret	45.617013	26.002523	747	CarE-O-E	Siret			4	4		4	4
Romania	E Carpathians	Putna	Siret	45.924523	26.713035	368	CarE-O-E	Siret			4	4		4	4
Romania	S Carpathians	Tarlung	Râul Negru	45.552172	25.785739	541	CarS-O-S	Râul Negru, Olt & Prahova		4	4	4		4	4
Romania	S Carpathians	Ogretineanca	Prahova	45.241031	26.078167	398	CarS-O-S	Râul Negru, Olt & Prahova	4	4		4			
Romania	S Carpathians	Prahova	Prahova	45.329660	25.565942	756	CarS-O-S	Râul Negru, Olt & Prahova							4
Romania	S Carpathians	Taleajen	Prahova	45.364777	25.933967	680	CarS-O-S	Râul Negru, Olt & Prahova						3	
Romania	S Carpathians	Șușița	Jiu	45.050000	23.240000	212	CarS-O-W	Jiu, Belareca & Cerna		3	3				4
Romania	S Carpathians	Brebina	Jiu	44.996774	22.827452	266	CarS-O-W	Jiu, Belareca & Cerna				3	3		
Romania	S Carpathians	Sohodol	Jiu	45.086789	23.145263	243	CarS-O-W	Jiu, Belareca & Cerna				4			
Romania	S Carpathians	Jiu	Jiu	45.095000	23.317000	234	CarS-O-W	Jiu, Belareca & Cerna		5	5			3	3
Romania	S Carpathians	Belareca	Cerna	44.893000	22.372000	177	CarS-O-W	Jiu, Belareca & Cerna		3				4	3
Romania	S Carpathians	Temes	Temes	45.487000	22.185000	174	CarC-I-S	Temes & Mureș			5	4	3	4	4
Romania	S Carpathians	Strei	Mureș	45.474000	23.160000	470	CarC-I-S	Temes & Mureș		4	4	4	4	3	5
Romania	Apuseni	Râul Arieș	Mureș	46.503000	23.608000	364	CarC-I-S	Temes & Mureș		3	3	5		3	5
Romania	Apuseni	Bega	Tisza	45.831000	22.021000	124	CarC-I-A	Tisza	4	3					
Romania	Apuseni	Crişul Repede	Tisza	46.893000	22.877000	499	CarC-I-A	Tisza	2	3		5	5	4	
Romania	Apuseni	Crişul Alb	Tisza	46.182000	22.665000	240	CarC-I-A	Tisza	4	3		3	2	3	5
Romania	Apuseni	Crişul Negru	Tisza	46.597000	22.416000	209	CarC-I-A	Tisza		4	3	3	5	4	3

We used 757 individuals: 111 (Bvar), 126 (Bdec), 117 (Bpun), 72 (Bmod), 118 (Prub), 105 (Pruf), 108 (Plim), from 19, 31, 28, 32, 29, 27 and 30 sites, respectively.

Individuals were immediately preserved in 96% ethanol and further stored at -22ºC for molecular use. Before DNA extraction, each individual was determined to the species level.

### Molecular analyses

#### Laboratory procedures

Whole beetle bodies were used for DNA extraction using a Nucleospin Tissue kit (Macherey–Nagel). *Cox1* was amplified using primer pairs: B1490-Bcoi2R^37^, LCO1490-HCO2198^50^ or Paed-F2-Paed-R2^51^. Nuclear protein-coding gene – Arginine Kinase (*ArgK*) was amplified using primers AK168F and AK939R^52^. The reagent concentrations used for the amplifications and PCR cycling profiles of both markers were as in Kolasa et al.^51^. After purification, the PCR fragments were sequenced using a BigDye Terminator v.3.1. Cycle Sequencing Kit (Applied Biosystems) and run on an ABI 3100 Automated Capillary DNA Sequencer. Sequences (haplotype only) are deposited in GenBank under accession numbers provided in Appendix 1.

#### Species assignment confirmation

Prior to intraspecific analyses, all newly generated sequences were compared with available resources in GenBank using Basic Local Alignment Search Tool (https://blast.ncbi.nlm.nih.gov/)^53^. All sequences occurred to belong to the species assigned to the species based on their morphology (data not shown). Moreover, all newly generated sequences were aligned with selected sequences of other *Bembidion* or *Paederus/Paederidus* species, and maximum-likelihood trees were generated using IqTree server (http://iqtree.cibiv.univie.ac.at/)^54^. In all cases, sequences were grouped consistently according to the morphological identification of beetles (all sequences from particular species formed a monophyletic group) (data not shown).

### Statistics

Sequences were aligned in MAFFT v. 7^55^. Aligned sequences were trimmed and translated into protein sequences in MEGA11 to check against pseudogenes^56^. For some analyses, sequences were grouped according to their geographic provenance. Grouping was based on regions of the Carpathians (W. Carpathians vs. E. Carpathians vs. S. Carpathians vs. Apuseni Mts.) or according to river basins (Table 2).

Standard genetic indices for populations such as haplotype number (*H*), haplotype diversity (*Hdiv*), nucleotide diversity (*πdiv*), as well as inter-population indices like *F*_*ST*_, *G*_*ST*_ and *N*_*ST*_ were computed using the program DnaSP v. 5^57^, separately for both markers. The values of the latter indices were next shown in matrix heathmaps considering the geographic groups according to Table 2 and with use of Heatmapper (http://www.heatmapper.ca).

To check which portion of genetic variation was present between populations, an analysis of molecular variance (AMOVA) was conducted in ARLEQUIN v. 3.5^58^.

An isolation by distance (IBD)^59^ was performed in ARLEQUIN v. 3.5^58^, using pairwise F_ST_ values (F_ST_ / (1-F_ST_)) and straight-line geographic distances in kilometers (log(km)).

### Haplotype networks and maps

Neighbor-joining (NJ) haplotype networks^60^ were reconstructed separately for each marker in PopArt^61^, and the same was also done for the combined sequences. Distribution of haplotypes over the Carpathians (separately for each marker and species) was visualized using QGIS ver. 3.28.9 (http://qgis.org) and CorelDraw ver. 18 (http://coreldraw.com). All maps were created using QGIS based on elevation raster downloaded from the WorldClim database (http:/www.worldclim.org/data/worldclim21.html;^62^) and shapefile "Hydrography of Europe", downloaded from http://www.efrainmaps.es. Carlos Efraín Porto Tapiquén. Geografía, SIG y Cartografía Digital. Valencia, Spain, 2020. All source files used for visualization of data are freely available.

### Demographic estimates

Demographic analyses were done solely on *Cox1* due to lacking reliable estimates of mutation rates for the *ArgK* gene in beetles.

Two statistical tests Tajima^63^ D test and Fu^64^ Fs were used for verifying neutrality of examined sequences and to detect signs of past demographic changes in the examined populations.

A mismatch distribution (MD)^65^ was calculated in ARLEQUIN v. 3.5 only for *Cox1* in order to examine the demographic history, and specifically, test for historical (temporal) expansions of populations of the species. The probable time of expansion (how long ago the expansion occurred) was estimated by the parameter τ. Due to lack of calibration events based on (sub)fossil materials for examined species, the mutation rate was estimated in range of 0.0158–0.0196 divergence lineages per million years according to Papadopoulou et al.^66^.

Additionally, a coalescent-based Bayesian skyline plot (BSP) was generated using BEAST v. 2.6.5^67^. To investigate the posterior probability distribution of effective population size (*Ne*), the best-fit model of nucleotide substitutions and their parameter values as priors were estimated without the assumption of any particular demographic model^68^. The *Cox1* and *ArgK* nucleotide substitution rates were set to the average value commonly found in beetles^65^ with a strict molecular clock. Markov chain Monte Carlo sampling was run for 10 million generations with parameters sampled for every 5.0 × 10^4^ generation. The initial 10% of the run was discarded as burn-in. We determined the effective sample size for the posterior distribution of estimated parameter values using Tracer v. 1.6^69^.

### Supplementary Information

Supplementary Information 1.

Supplementary Information 2.

Supplementary Information 3.

Supplementary Information 4.

Supplementary Information 5.

## Data Availability

All data collected to this study are freely available: DNA sequences (haplotype only) are deposited in GenBank under accession numbers: *Cox1*: Bdec OQ176441-OQ176476, Bmod OQ176496-OQ176515, Bpun OQ176477-OQ176495, Bvar OQ176516-OQ176531, Plim OQ176532-OQ176566, Prub OQ176602-OQ176664, Pruf OQ176690-OQ176728, ArgK: Bdec OQ197683-OQ197703, Bmod OQ197704-OQ197719, Bpun OOQ197720-OQ197746, Bvar OQ197747-OQ197767, Plim OQ197768-OQ197815, Prub OQ197816-OQ197832, Pruf OQ197833-OQ197851.
