# Supplementary material for: Limited congruence in phylogeographic patterns observed for riverine predacious beetles sharing distribution along the mountain rivers
Source: Sci Rep. 2023 Oct 19;13:17883. doi: 10.1038/s41598-023-44922-w (PMC10587157; doi:10.1038/s41598-023-44922-w)
Supplement: Supplementary file 1 — Supplementary Information 1. [file 41598_2023_44922_MOESM1_ESM.docx]

**Appendix 1.** GenBank under accession numbers:

Cox1

*B.decorum* OQ176441-OQ176476

*B. modestum* OQ176496-OQ176515

*B. punctulatum* OQ176477-OQ176495

*B. varicolor* OQ176516-OQ176531

*P. limnophilus* OQ176532-OQ176566

*P. rubrothoracicus* OQ176602-OQ176664

*P. ruficollis* OQ176690-OQ176728

ArgK

*B. decorum* OQ197683-OQ197703

*B. modestum* OQ197704-OQ197719

*B. punctulatum* OQ197720-OQ197746

*B. varicolor* OQ197747-OQ197767

*P. limnophilus* OQ197768-OQ197815

*P. rubrothoracicus* OQ197816-OQ197832

*P. ruficollis* OQ197833-OQ197851
