# Supplementary figures and images for: Limited congruence in phylogeographic patterns observed for riverine predacious beetles sharing distribution along the mountain rivers
Source: Sci Rep. 2023 Oct 19;13:17883. doi: 10.1038/s41598-023-44922-w (PMC10587157; doi:10.1038/s41598-023-44922-w)

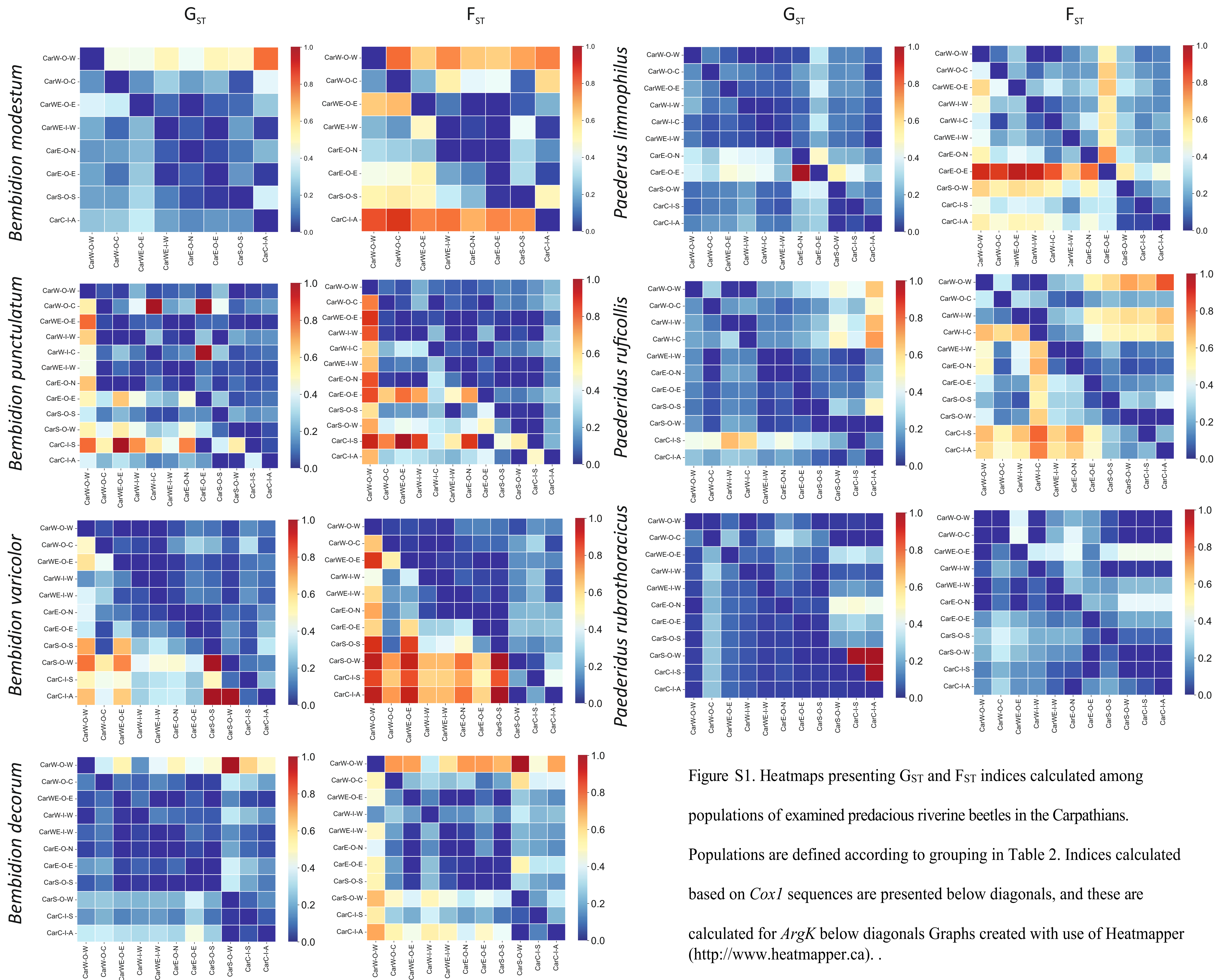

Supplement: Supplementary file 2 — Supplementary Information 2. [file 41598_2023_44922_MOESM2_ESM.pdf]
