# Supplementary material for: Limited congruence in phylogeographic patterns observed for riverine predacious beetles sharing distribution along the mountain rivers
Source: Sci Rep. 2023 Oct 19;13:17883. doi: 10.1038/s41598-023-44922-w (PMC10587157; doi:10.1038/s41598-023-44922-w)
Supplement: Supplementary file 3 — Supplementary Information 3. [file 41598_2023_44922_MOESM3_ESM.pdf]

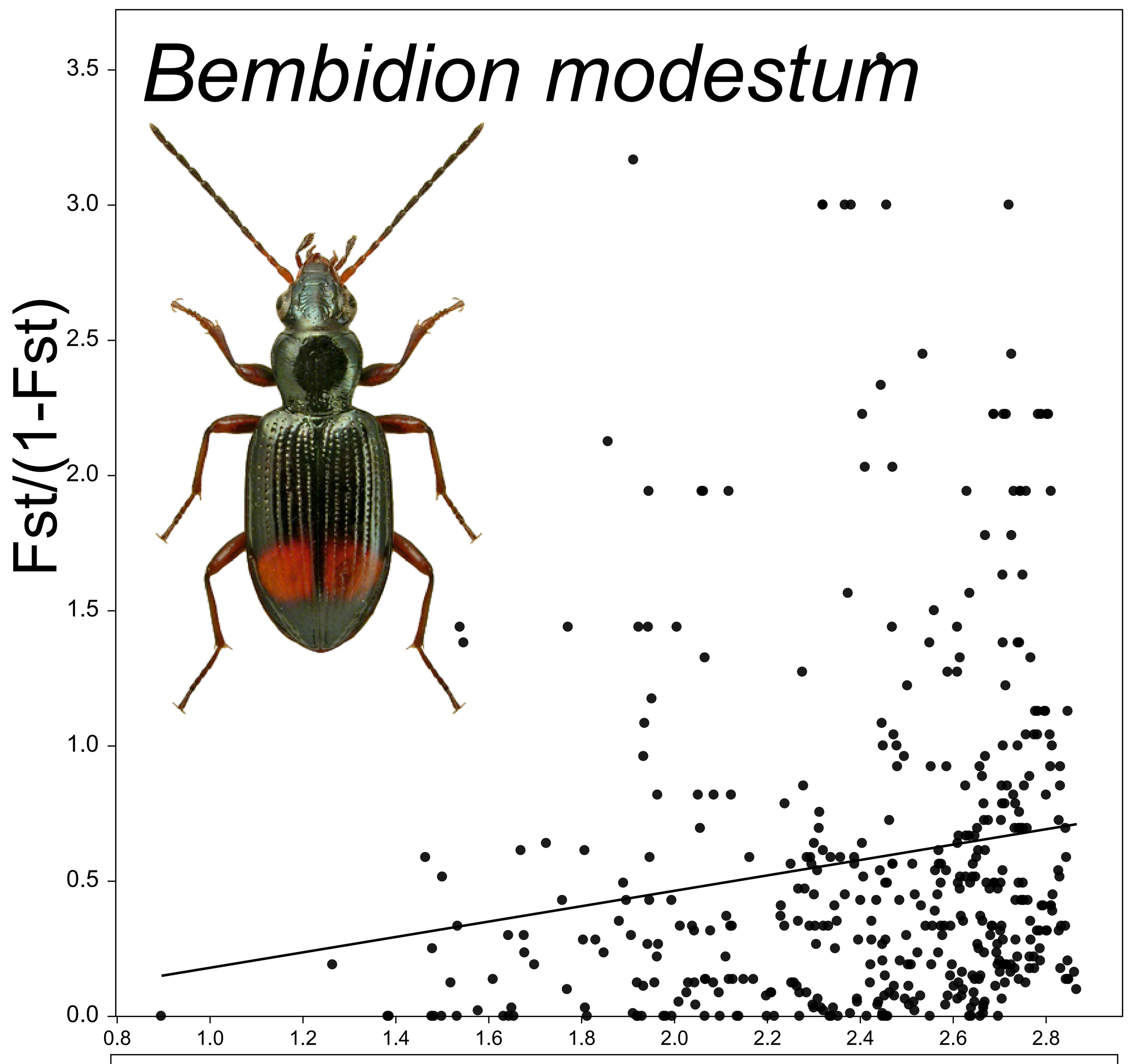

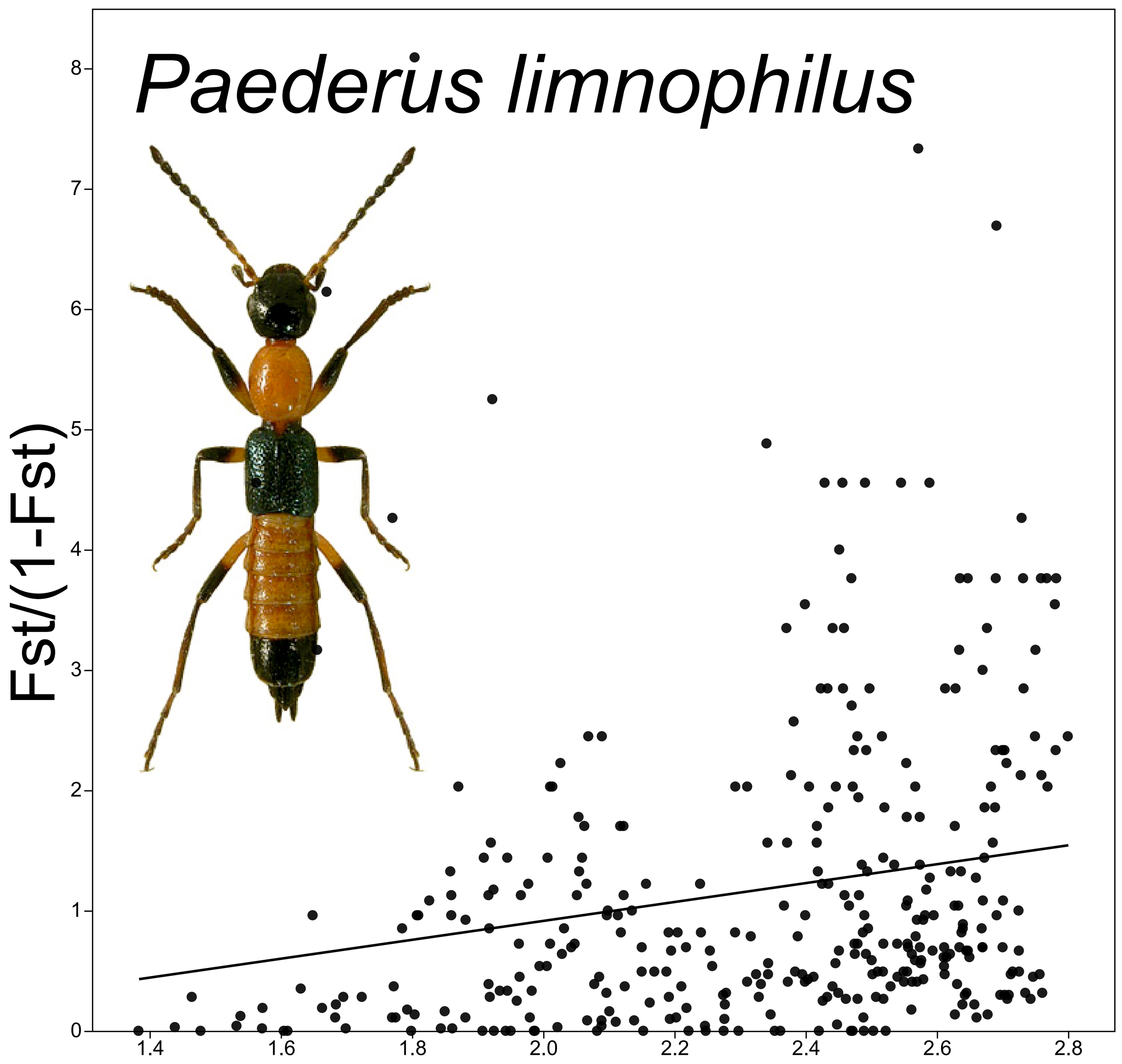

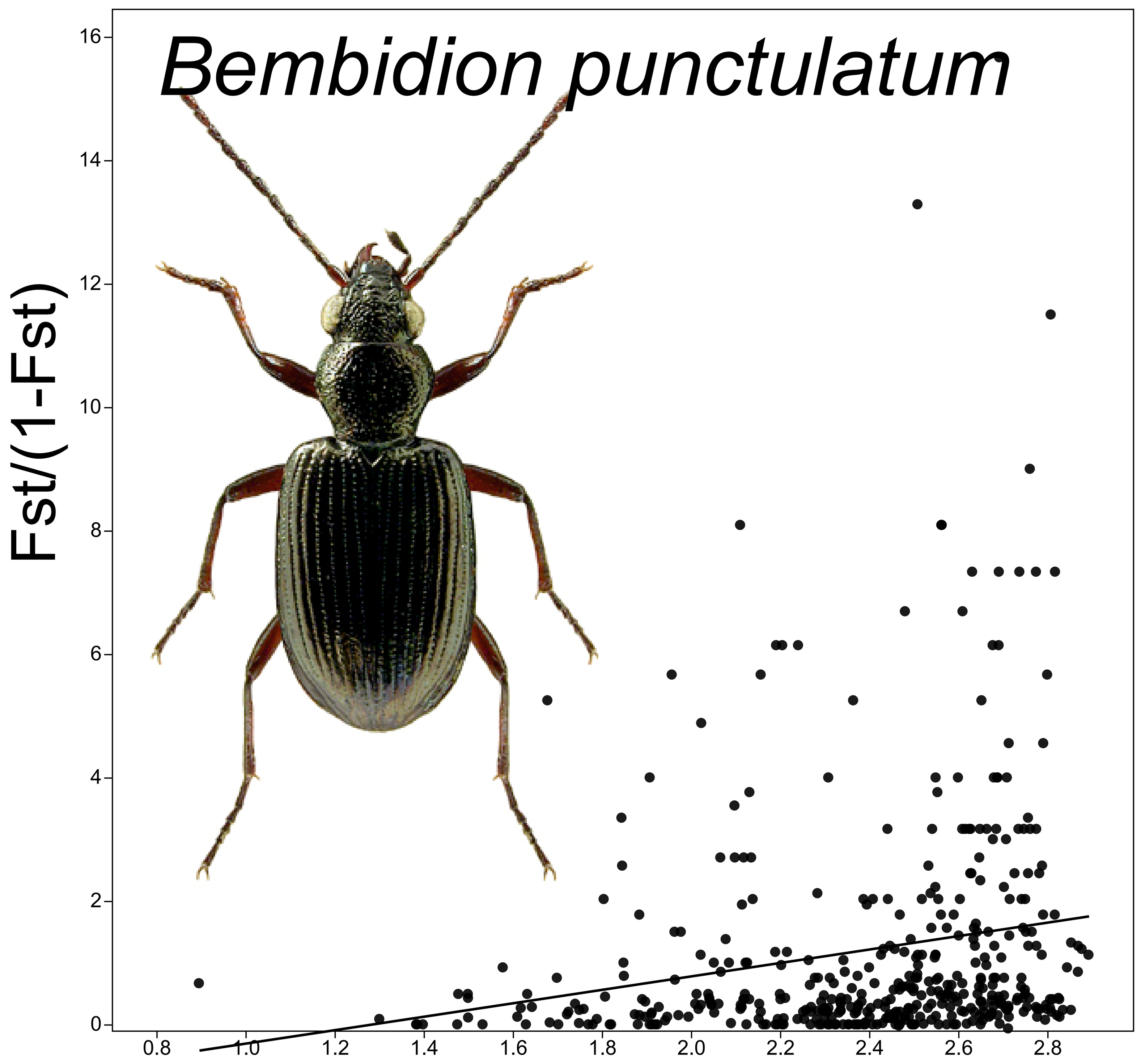

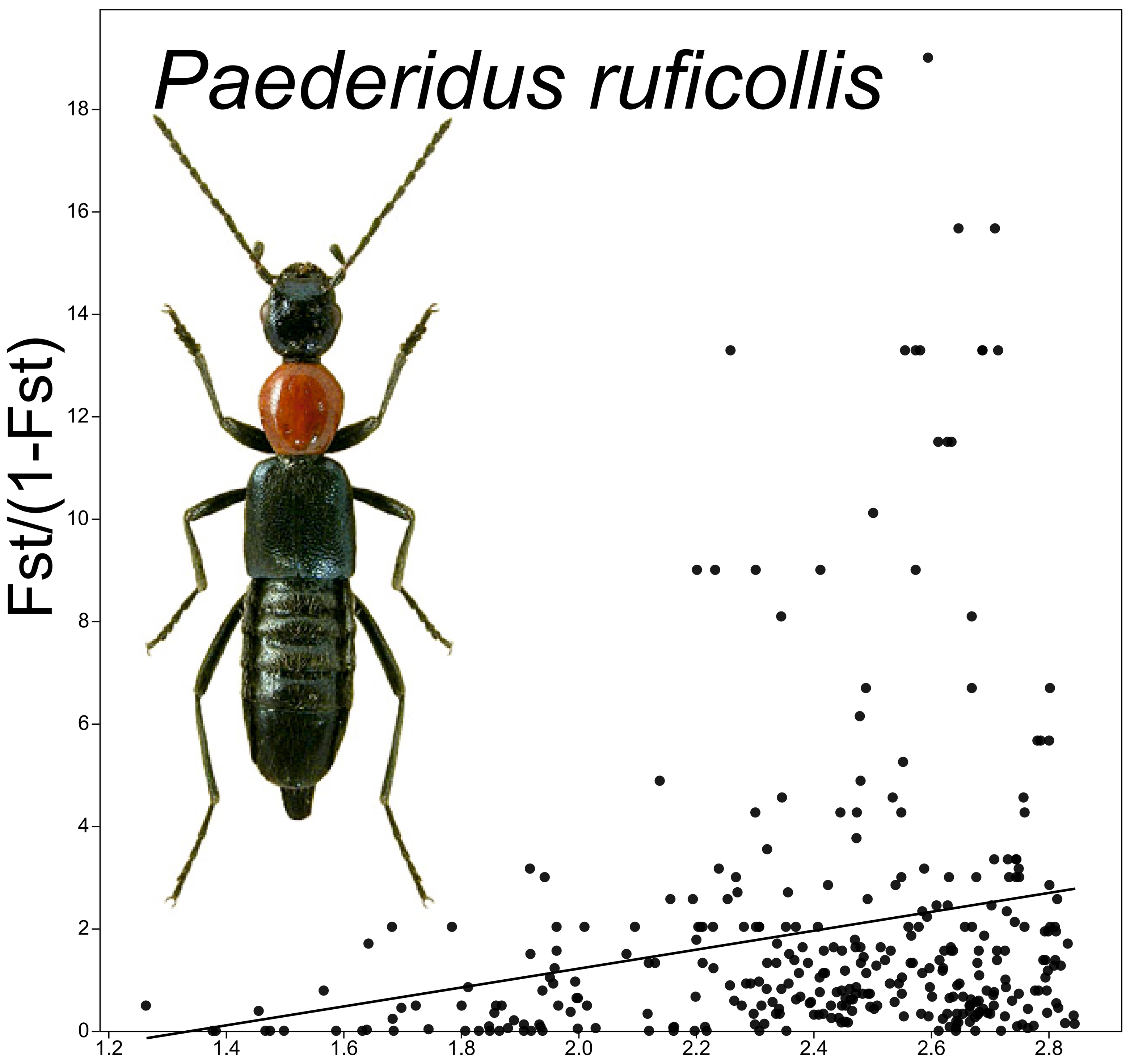

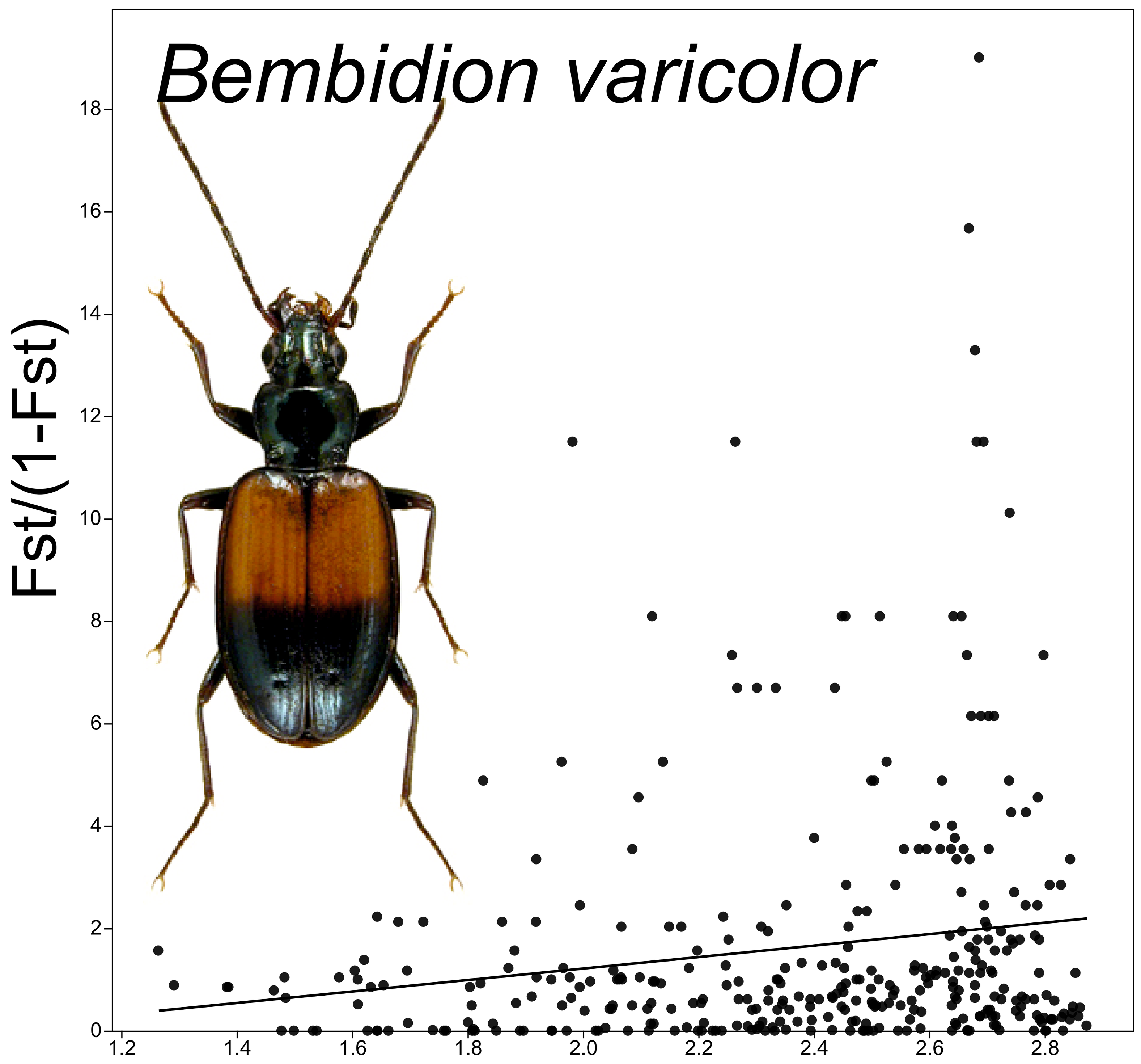

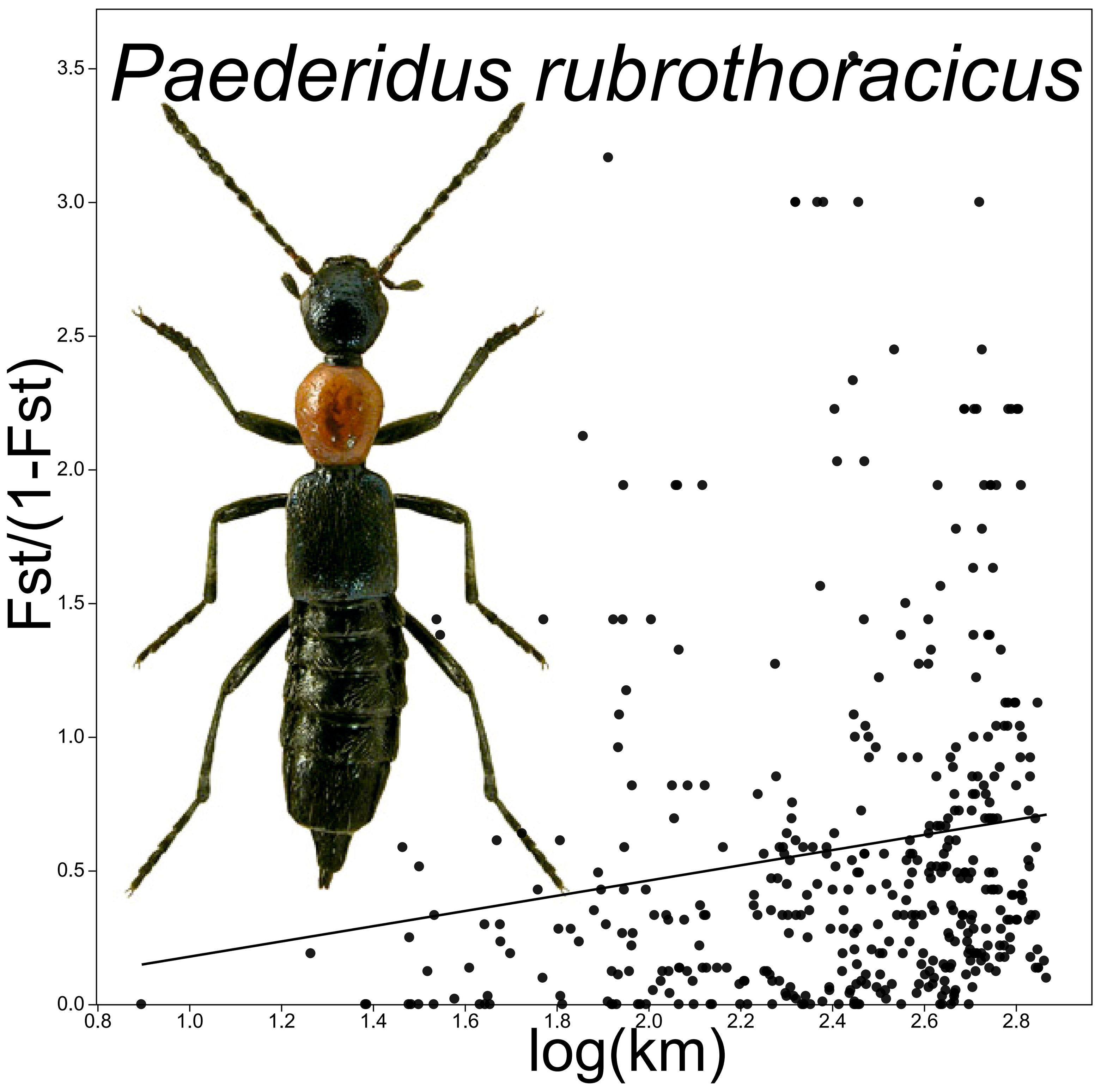

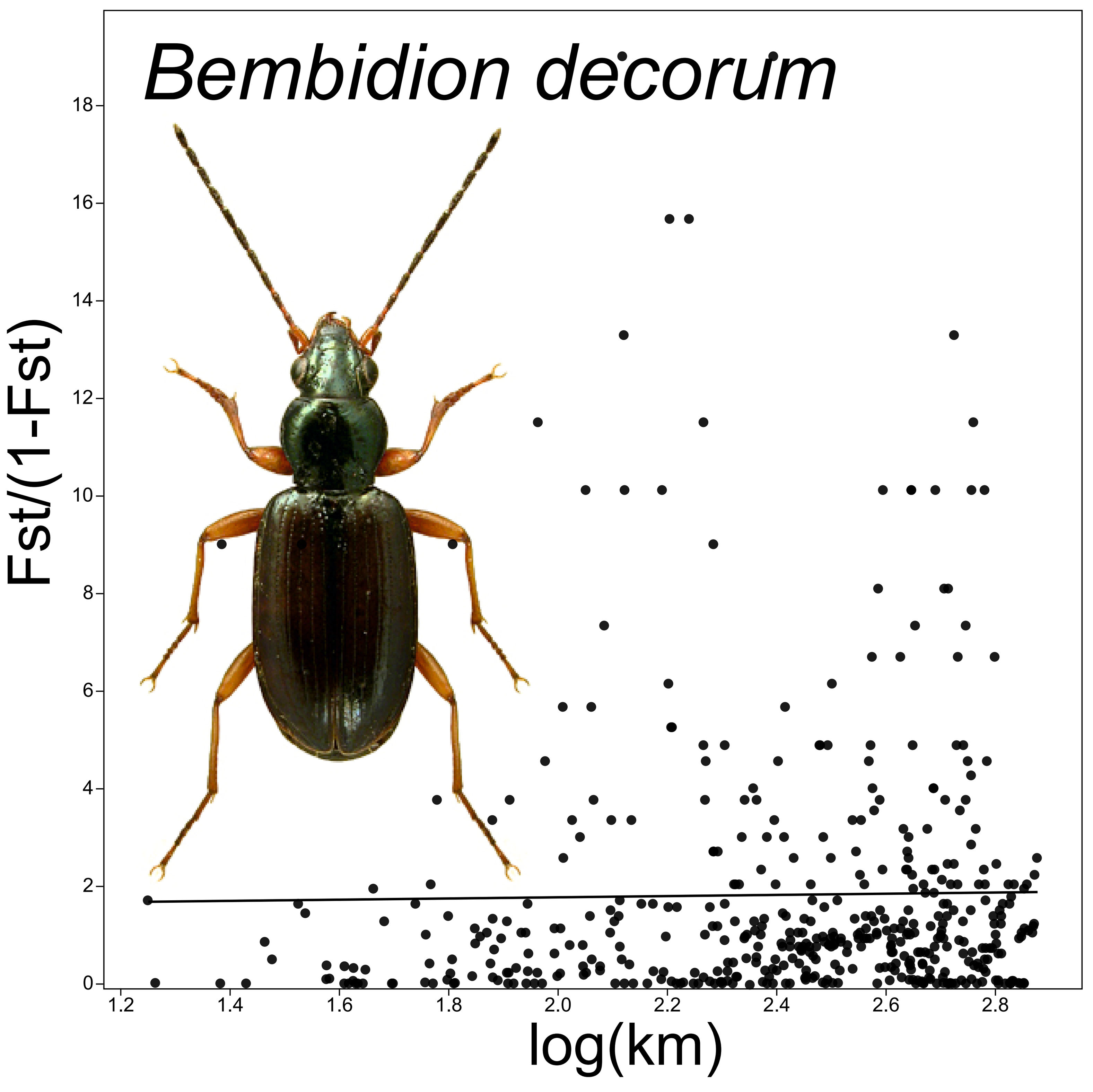

Figure S2. Plots of correlation between geographic distance (log(km) and genetic distance (Slatkin F_{ST}) measured among populations of examined predacious riverine beetles in the Carpathians.

Photographs of beetles are from ICONOGRAPHIA COLEOPTERORUM POLONIAE (© Copyright by Prof. Lech Borowiec, Wrocław 2007 –2014, Department of Biodiversity and Evolutionary Taxonomy, University of Wroclaw, Poland).
