# Supplementary material for: Limited congruence in phylogeographic patterns observed for riverine predacious beetles sharing distribution along the mountain rivers
Source: Sci Rep. 2023 Oct 19;13:17883. doi: 10.1038/s41598-023-44922-w (PMC10587157; doi:10.1038/s41598-023-44922-w)
Supplement: Supplementary file 4 — Supplementary Information 4. [file 41598_2023_44922_MOESM4_ESM.docx]

**Table S1.** Analysis of Molecular Variance for examined predacious riverine beetles in the Carpathians.

| AMOVA / species | *B. modestum* | *B. punctulatum* | *B. varicolor* | *B. decorum* | *P. limnophilus* | *P. ruficollis* | *P. rubrothoracicus* |
| --- | --- | --- | --- | --- | --- | --- | --- |
| grouping according 4 regions | | | | | | | |
| (W Carpathians vs E Carpathians vs S Carpathians vs Apuseni) | | | | | | | |
| Within populations | 27 | 59 | 49 | 46 | 47 | 33 | 68 |
| Among populations within groups | 39 | 35 | 34 | 42 | 31 | 34 | 23 |
| Among groups | 34 | 6 | 17 | 12 | 22 | 33 | 9 |
| Σ | 100 | 100 | 100 | 100 | 100 | 100 | 100 |
| F_SC_ | 0.59 | 0.37 | 0.41 | 0.48 | 0.41 | 0.50 | 0.26 |
| F_ST_ | 0.72 | 0.41 | 0.51 | 0.54 | 0.53 | 0.67 | 0.32 |
| F_CT_ | 0.34 | 0.06 | 0.63 | 0.12 | 0.22 | 0.33 | 0.09 |
| grouping according river basins | | | | | | | |
| Within populations | 29 | 59 | 52 | 47 | 20 | 46 | 69 |
| Among populations within groups | 27 | 19 | 27 | 33 | 30 | 19 | 18 |
| Among groups | 44 | 22 | 21 | 20 | 50 | 35 | 13 |
| Σ | 100 | 100 | 100 | 100 | 100 | 100 | 100 |
| F_SC_ | 0.48 | 0.24 | 0.34 | 0.41 | 0.38 | 0.29 | 0.21 |
| F_ST_ | 0.71 | 0.40 | 0.47 | 0.53 | 0.50 | 0.54 | 0.32 |
| F_CT_ | 0.44 | 0.21 | 0.21 | 0.20 | 0.20 | 0.35 | 0.13 |
