# Supplementary material for: Limited congruence in phylogeographic patterns observed for riverine predacious beetles sharing distribution along the mountain rivers
Source: Sci Rep. 2023 Oct 19;13:17883. doi: 10.1038/s41598-023-44922-w (PMC10587157; doi:10.1038/s41598-023-44922-w)
Supplement: Supplementary file 5 — Supplementary Information 5. [file 41598_2023_44922_MOESM5_ESM.docx]

**Table S2.** Statistics describing demography of examined predacious riverine beetles in the Carpathians.

| species | Tajima's | | Fu's | | Mismatch Distribution | |
| --- | --- | --- | --- | --- | --- | --- |
|  | D | p | Fs | p | Theta | Tau |
| *B. modestum* | -1,32562 | > 0.10 | -9,129 | <0.001 | 1.852 | 1.079 |
| *B. punctulatum* | -2,24206 | < 0.01 | -26,84 | <0.001 | 0.663 | 1.113 |
| *B. varicolor* | -1,11788 | > 0.10 | -20,076 | <0.001 | 0.000 | 1.360 |
| *B. decorum* | -1,48311 | > 0.10 | -12,201 | <0.001 | 0.000 | 2.035 |
| *P. limnophilus* | -1,97146 | < 0.05 | -33,141 | <0.001 | 3.453 | 1.027 |
| *P. ruficollis* | -1,21959 | > 0.10 | -14,634 | <0.001 | 1.110 | 1.865 |
| *P. rubrothoracicus* | -1,56564 | <0.10 | -13,463 | <0.001 | 3.760 | 3.147 |
